# Case of Congenital Cataract in Both Eyes, One Operated on Successfully

**Published:** 1845-11

**Authors:** George Dock

**Affiliations:** Harrisburg, Pennsylvania


*Case of Congenital Cataract in both eyes, one operated on successfully.* By George Dock, M. D., of Harrisburg, Pennsylvania. Communicated in a letter to the editor.

Dear Sir,—Since my return from Philadelphia, I was sent for by Dr. William Henderson, to see the daughter of Mr. John Balsbaugh, residing eleven miles from this place. The little girl was about three years of age, and a fine, healthy, intelligent child, but with perfectly formed cataract in both eyes, and which on inquiry proved to have been congenital. As the constitution of the child was favourable, the eyes healthy, well formed and handsome, the pupil well dilated and free from adhesions all around, with the consent of the parents and concurrence of the family physician, I determined to operate on one eye first, waiting to see the result before proceeding further. Having prepared the patient that day, I next morning applied belladonna around the right eye, which by two o'clock had fully dilated the pupil, when I proceeded to operate in the following manner:

Having placed the patient in the lap of a gentleman, with its back bearing against his breast, with his arms around its body and over its arms, so as to keep them down, I took a seat on the right and a little behind, and with my thumb and fore finger of the left hand drew up the upper lid, whilst an assistant, Dr. Shope, depressed the lower lid with his finger. Having thus dispensed with a speculum, (which indeed I never use) I introduced a small straight needle into the sclerotica about a line and a half from the cornea, carried it above the lens, and finding it to be a soft capsulo-lenticular cataract, divided the lens and capsule from above downwards, before backwards, &c., disengaging the capsule well from the ciliary body so as to leave nothing to form a secondary cataract, (of which I have seen several) caused by the remaining part of the capsule floating about the pupil and obstructing the rays of light. Being one of those soft cataracts which are so troublesome to keep down after couching them, it rose several times, but at length I made a bed for it in the vitreous humour and kept it there with my needle several seconds, when, with a gentle rotary motion, I disengaged the instrument and withdrew it. The pupil was clear and beautiful. I closed the eye, threw a light bandage around, and placed the child in its mother's arms in a darkened room, and remained in the house myself during the night. In the morning, finding every thing going on well, I left, with a request to be informed of the progress of the case. Three or four days after the operation, I received a letter from Dr. Henderson saying that the conjunctiva was perfectly clear and healthy, that the eye looked beautiful, and that there had not been the least discoverable enlargement of the vessels in the neighborhood of the wound caused by the needle. The patient is now so well as to render it probable that I shall soon be able to operate on the other eye.

This is the third case of congenital cataract I have operated on within the past ten months, and all with the same complete success, in none of which has there been so much inflammation as to require a purge.

*Harrisburg, September,* 1845.

